# Above-Standard Survival of Hepatocellular Carcinoma as the Final Outcome of Comprehensive Hepatology Care Programs in a Remote HCV-Endemic Area

**DOI:** 10.3390/v15030786

**Published:** 2023-03-19

**Authors:** Wei-Ru Cho, Hui-Ling Huang, Nien-Tzu Hsu, Tung-Jung Huang, Te-Sheng Chang

**Affiliations:** 1 Department of Hepatology and Gastroenterology, Division of Internal Medicine, Chang Gung Memorial Hospital, Yunlin 638502, Taiwan; 2 Department of Nursing, Chang Gung Memorial Hospital, Chiayi 613016, Taiwan; 3 Department of Information Management, National Chung Cheng University, Chiayi 621301, Taiwan; 4 Biostatistics Center of Kaohsiung Chang Gung Memorial Hospital, Kaohsiung 833253, Taiwan; 5 Department of Thoracic Medicine, Division of Internal Medicine, Chang Gung Memorial Hospital, Yunlin 638502, Taiwan; 6 Department of Hepatology and Gastroenterology, Division of Internal Medicine, Chang Gung Memorial Hospital, Chiayi 613016, Taiwan; 7 College of Medicine, Chang Gung University, Taoyuan City 333323, Taiwan

**Keywords:** hepatocellular carcinoma, referral, screening

## Abstract

Early detection and prompt linkage to care are critical for hepatocellular carcinoma (HCC) care. Chang Gung Memorial Hospital (CGMH) Yunlin branch, a local hospital in a rural area, undertakes health checkup programs in addition to its routine clinical service. Patients with HCC are referred to CGMH Chiayi branch, a tertiary referral hospital, for treatment. This study enrolled 77 consecutive patients with newly diagnosed HCCs between 2017 and 2022, with a mean age of 65.7 ± 11.1 years. The screening group included HCC patients detected through health checkups, and those detected by routine clinical service served as the control group. Compared to the 24 patients in the control group, the 53 patients in the screening group had more cases with early stage cancer (Barcelona Clinic Liver Cancer or BCLC stage 0 + A 86.8% vs. 62.5%, *p* = 0.028), better liver reserve (albumin–bilirubin or ALBI grade I 77.3% vs. 50%, *p* = 0.031) and more prolonged survival (*p* = 0.036). The median survival rates of the 77 patients were >5 years, 3.3 years, and 0.5 years in the BCLC stages 0 + A, B, and C, respectively, which were above the expectations of the BCLC guideline 2022 for stages 0, A, and B. This study provides a model of HCC screening and referral to high-quality care in remote viral-hepatitis-endemic areas.

## 1. Introduction

Hepatocellular carcinoma (HCC) is the fifth most common cancer worldwide and the second most frequent cause of cancer-related deaths [1,2]. HCC and its complications place a significant burden on public health, the healthcare system, and the economy globally. In 2020, the crude incidence rate of HCC was 46.61 per 100,000 person-years, the fifth highest among all cancers in Taiwan and the crude mortality rate was the second highest among all cancers, 32.99 per 100,000 person-years. Factors such as hepatitis B virus (HBV) or hepatitis C virus (HCV) infections, alcohol abuse, metabolic syndrome (particularly non-alcoholic fatty liver disease) and exposure to dietary toxins such as aflatoxins are considered to be related to the development of hepatocellular carcinoma [3]. The above risk factors are potentially preventable, highlighting that risk prevention can decrease the burden of HCC [4]. Furthermore, regular surveillance is associated with early HCC detection and might increase the chance of potentially curative treatment [5].

In Taiwan, the primary cause of HCC is HBV infection, followed by HCV infection, with similar observations reported in most areas of Asia and Sub-Saharan Africa [5,6,7]. Due to chronic carriers of hepatitis viruses usually being asymptomatic, most people remain unaware of their HBV or HCV infection and their infection will develop into more advanced diseases after several decades [8]. As a result, the incidence and mortality of HCC in the Taiwanese population remain high and are worthy of attention, especially in rural and remote communities. With this in mind, screening and the early identification of asymptomatic people with chronic HBV or HCV infection became an important issue in Taiwan. This important health problem caught the attention of the government and hepatologists. Health scientists and doctors have become devoted to finding those who need to receive antiviral treatment for HBV or HCV to improve their health status and to also provide these patients with the chance for early detection and efficient treatment of HCC for a favorable outcome [5,8,9]. 

Some major advances in HCC management strategies have facilitated the experts to update of the Barcelona Clinic Liver Cancer (BCLC) guideline in 2022 to guide clinical decision-making for HCC worldwide [9]. Despite the great progress in the treatment of HCC, the overall prognosis is still unsatisfactory, and efforts are needed to overcome this dilemma. For remote and rural communities that typically face difficulties accessing medical resources, screening strategies to identify asymptomatic people living with hepatitis B or C, to diagnose patients with HCC early, and efficaciously refer individuals eligible for treatment, constitute a crucial component of HCC care [9]. To achieve an ideal outcome of HCC for remote and rural communities, several challenges exist, including sustainable financing, practical and efficient screening programs, the continuum of treatment and care, and improving accessibility [10].

Under the feedback funding programs of a local petrochemical corporation, Chang Gung Memorial Hospital Yunlin Branch (YLCGMH) cooperated with the Nursing Department of Chiayi Chang Gung University of Science and Technology to execute health checkups and provided post-examination care. The YLCGMH is a 100-bed local hospital with facilities for almost all laboratory tests, and radiological items, but it has only limited facilities for HCC treatment. Therefore, patients with newly diagnosed or recurrent HCC by either health checkups (screening group) or routine clinical services (non-screening group) in YLCGMH were transferred to the CGMH Chiayi branch (CYCGMH), a tertiary referral hospital with full facilities for HCC treatment. We conducted this retrospective study to evaluate the differences of HCC survival between screening and non-screening patient populations. We also compared the HCC survival result of our comprehensive hepatology care program for rural communities with the expectations of the updated BCLC guideline 2022 [9,11].

## 2. Patients and Methods

### 2.1. Background of Study Area

**Figure 1:**
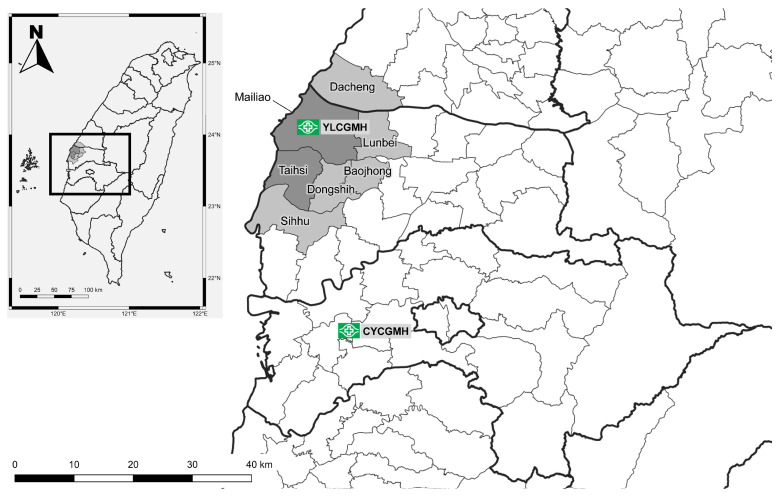
Screening and linkage to accessible care.

Mailiao and Taihsi are two neighboring rural townships located in the central west coastal area of Taiwan, with a combined population of 77,110 registered residents in 2022. A previous study revealed the prevalence of anti-HCV reached as high as 55% among adult residents in the Taihsi Township [12]. Furthermore, the prevalence of anti-HCV for male HCC patients in the Mailiao Township was more than 50% in a large cohort study [13]. These two townships were reported to have the highest liver cancer mortality age-standardized rates (ASRs) up to 80.43 and 83.47 per 100,000 person-years in the Yunlin county [14]. The YLCGMH is the closest local hospital to these two townships, both with an exceptionally high prevalence of chronic viral hepatitis B and C patients [15]. As a local hospital, the routine daily medical work of YLCGMH includes multidisciplinary outpatient, inpatient and emergency services. This local hospital works to improve their patients’ health from nearby townships in coordination with the Nursing Department of Chiayi Chang Gung University of Science and Technology [12,16,17,18,19,20]. In addition to annual checkups for Mailiao and Taihsi, outreach health checkups, including liver disease screening, were also conducted in five surrounding townships (Sihhu, Baojhong, Dacheng, Dongshih and Lunbei Townships), as depicted in Figure 1 The outreach health checkups were conducted in up to sixty-five villages between 2018 and 2022. Villagers positive for anti-HCV or HBsAg were called back for further evaluation and treatment [17]. Regular shuttle services between YLCGMH and CYCGMH and certain townships were also provided to improve medical accessibility.

### 2.2. Patients 

Sponsored since 2012 by a local petrochemical corporation, all the registered residents in the Mailiao and Taihsi Townships were eligible for, and were encouraged to participate in, the annual health checkups without any restriction. Outreach health checkups for all inhabitants in another five surrounding townships were initiated in 2018. Items concerning hepatitis and HCC screening were included in these health checkup programs, i.e., hepatitis B virus surface antigen s(HBsAg), antibodies against HCV (anti-HCV), alpha-fetoproteins (AFP), and abdominal ultrasonography performed by hepatologists in YLCGMH. Patients with HBV and HCV were treated in this local hospital according to international guidelines. Participants of the health checkups and patients of routine clinical services diagnosed with HCC in YLCGMH were referred to CYCGMH for further treatment.

About 10,000 people joined the health checkup program every year, with screening coverage rates above 50% of the total population in these nearby townships. Until 2022, an estimated 94.7% of participants who were positive for anti-HCV antibodies came back for HCV RNA testing, and 95.2% of viremic HCV patients in these townships have been cured with either peginterferon-based therapy or direct-acting antiviral drugs and they were encouraged to undergo regular follow-up after treatment.

Our study included all patients with newly diagnosed HCC in YLCGMH from October 2017 to October 2022. This study was approved by the Institutional Review Board of Chang Gung Memorial Hospital.

### 2.3. Definition

The diagnosis of HCC was based on the criteria of the American Association for the Study of Liver Disease (AASLD) and the European Association for the Study of the Liver (EASL) [21,22] and confirmed by histological analysis, when available. The diagnosis of cirrhosis was based on any of the following modalities: liver histology; fibrosis-4 (FIB-4) index (>6.5); or the presence of clinical, radiological, endoscopic, or laboratory evidence of cirrhosis and/or portal hypertension.

### 2.4. Calculation of ALBI Grades

The albumin–bilirubin (ALBI) grade, taking into account the levels of albumin and bilirubin, was calculated as follows: linear predictor  =  (log10 bilirubin × 0.66) + (albumin × −0.085), where the units of bilirubin and albumin are μmol/l and g/l, respectively. The ALBI grades were stratified into the following three grades: grade I, ≤−2.60; grade II, −2.60 to ≤−1.39; and grade III, >−1.39, as reported previously [23].

### 2.5. Screening and Linkage to Accessible Care

The health checkup programs included screening tests for liver diseases, such as HBsAg, anti-HCV, aspartate aminotransferase (AST), alanine aminotransferase (ALT), AFP, and abdominal ultrasonography that was performed by hepatology specialists in YLCGMH. Individuals with highly suspected liver tumors detected in the health checkups were referred to the hepatology clinics of YLCGMH. A biweekly hepatology clinic composed of physicians and surgeons from CYCGMH established in October, 2017 accepted internal and external referrals of patients with uncommon or severe liver diseases, mostly hepatocellular carcinoma. CT and MRI scans of livers can be performed in YLCGMH for further liver tumor evaluation. Patients with newly diagnosed or recurrent HCC who needed specific HCC treatment were transferred to the CYCGMH, a tertiary referral center with full facilities for HCC treatment. There are regular shuttles between YLCGMH and CYCGMH to shorten the gap in medical access. After treatment, the patients can receive post-treatment care and follow-up in YLCGMH. 

### 2.6. Statistical Analysis

Statistical analyses were performed using the SPSS 23.0 statistical package (SPSS, Inc., Chicago, IL, USA). Quantitative variables were expressed as mean ± standard deviation (SD) or medians with interquartile ranges (IQR). The chi-square test and Fisher’s exact test were used to compare categorical variables; the *t*-test or Mann–Whitney U-test were used to compare continuous variables. The overall survival (OS) relationships between groups were analyzed using Kaplan–Meier survival curves and the log-rank test. A two-tailed *p*-value of < 0.05 was considered as statistically significant. 

## 3. Results

### 3.1. Baseline Characteristics of HCC Patients in the Screening and Control Groups

**Figure 2:**
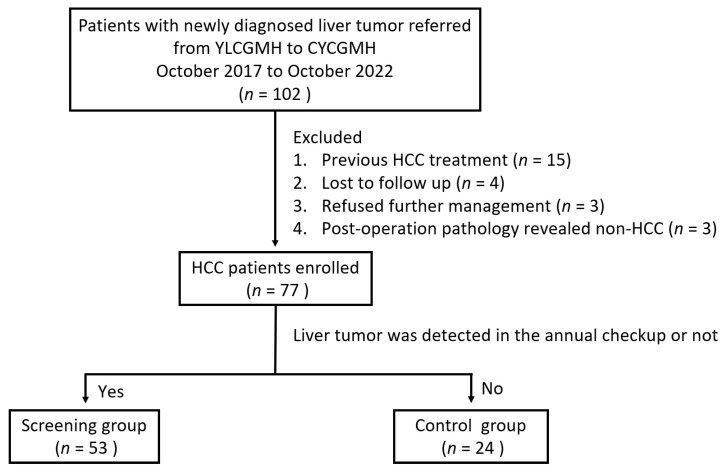
Patient enrollment flow diagram.

One hundred and two patients with liver tumors were referred from YLCGMH to CYCGMH during the study period. Of them, 25 patients were excluded, as 15 patients had received previous HCC treatment, 4 patients were lost to follow-up, 3 patients refused further management due to old age, and 3 patients were proved to be non-HCC after surgery. Finally, 77 patients with newly diagnosed HCC were enrolled in this study. They were further categorized into two groups based on whether the initial diagnosis of the liver tumor was detected in the annual health checkup. The screening group included 53 (68.9%) patients with their HCC detected in the annual health checkup, while the remaining 24 (31.2%) patients with their HCC detected in the routine clinical service served as the control group (Figure 2). 

Table 1 presents the baseline characteristics of the 77 HCC patients in this study cohort. There were 61 (79.2%) men and 16 (20.8%) women, with a mean age of 65.7 ± 11.1 years at enrollment. Of them, 66 patients were villagers in the Mailiao and Taihsi Townships. The risk factors of HCC were HBV (15/77; 19.4%), HCV (41/77; 53.2%), dual HBV and HCV (13/77; 16.8%), and non-HBV, non-HCV (NBNC) (8/77; 10.3%). The median level was 0.9 (IQR, 0.6–1.2) g/dL for total bilirubin, 4.2 (IQR, 3.9–4.6) g/dL for albumin, 45.9 (IQR, 26.0–55.5) U/L for AST, 43.5 (IQR, 21.5–59.0) U/L for ALT, and 8.4 (IQR, 3.6–54.3) ng/mL for AFP. Moreover, 73 patients belonged to Child–Pugh grade A, and 4 belonged to Child–Pugh grade B cirrhosis. In addition, there were 53, 23, and 1 patient with ALBI grade I, II, and III liver reserve, respectively. As for tumor stage, there were 21, 40 and 14 patients with BCLC stage 0, A and B, respectively, and 2 patients with BCLC stage C. Seventeen patients had more than one liver tumor. 

There were no statistically significant differences between the 53 patients in the screening group and the 24 patients in the control group regarding age (66 ± 11.0 vs. 65.3 ± 115, *p* = 0.809), gender (42:11 vs. 19:5, *p* = 0.994), distribution of viral hepatitis (NBNC:HBV:HCV: HBV + HCV 6:10:26:11 vs. 2:5:15:2, *p* = 0.523), Child–Pugh grade (A; B 52:1 vs. 21:3, *p* = 0.052) and tumor size in cm (2:2–5: > 5 18:33:2 vs. 9:11:4, *p* = 0.113). Patients in the screening group were 100% villagers in the Mailiao and Taihsi Townships; the control group included 11 patients (45.8%) who lived in other townships.

When compared to the control group, the screening group had a higher median level of albumin (4.3, 4.1–4.6 vs. 3.9, 3.4–4.6 g/dL, *p* = 0.040), lower ALT level (37.2, 24.5–44.5 vs. 65.2, 29–77.2 U/L, *p* = 0.044), more cases diagnosed in the early stage (BCLC stage 0 + A 86.8% vs. 62.5%, *p* = 0.028), better liver function reserve (ALBI grade I 77.3% vs. 50%, *p* = 0.031), and a higher percentage of single tumors (86.7% vs. 58.3%, *p* = 0.001). 

### 3.2. Antiviral Treaments for the Study Cohort 

Patients with HBV and HCV were treated in this local hospital according to international guidelines. There were 21 patients with detectable HBV DNA and 45 patients with detectable HVC RNA. All the patients with detectable HBV DNA received nucelos(t)ide analogues. Thirsty-three patients were treated with direct-acting antiviral agents and nine patients with peginterferon-based therapy among the patients with detectable HCV RNA. The SVR of the screening and control groups was as high as 86.2% and 92.3%, respectively.

### 3.3. Survival Outcome of the Study Cohort 

**Figure 3:**
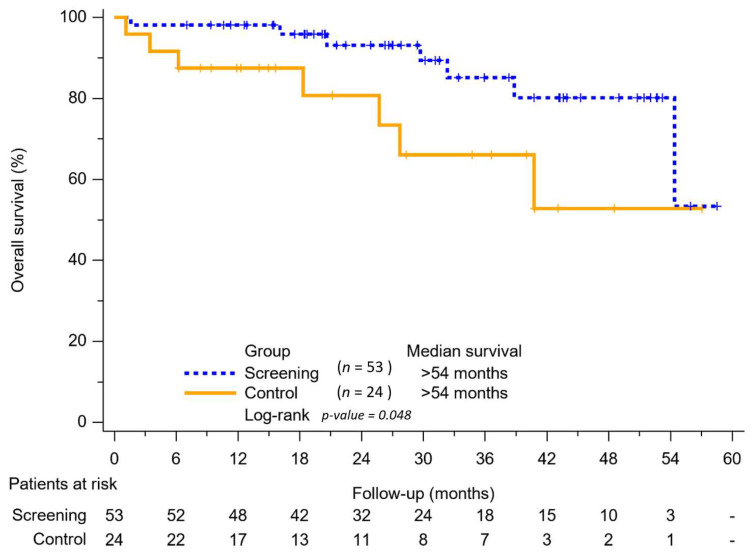
Kaplan–Meier analysis of overall survival between the screening group and control group.

As shown in Figure 3, the screening group had better overall survival (OS) than the control group, with a *p*-value of 0.048. More than half the patients among those two groups were still alive at the end of our study. Of the 77 patients, there were 21, 40, 14, and 2 patients with BCLC stage 0, A, B, and C, respectively. 

**Figure 4:**
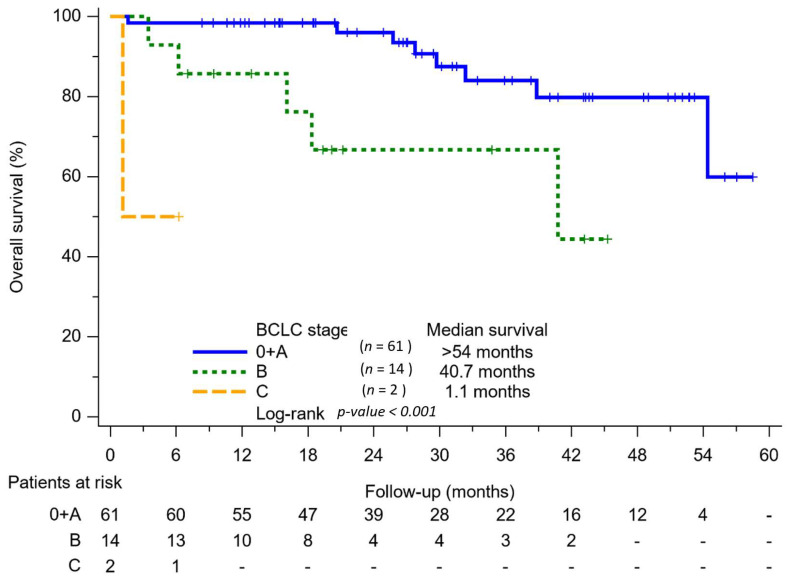
Kaplan–Meier analysis of overall survival among BCLC 0 + A, BCLC B and BCLC C patients.

The Kaplan–Meier analysis revealed that the BCLC 0/A patients had a higher OS than the BCLC B and BCLC C patients (*p* < 0.001) (Figure 4). In this 5-year follow-up study, more than half of the BCLC 0/A patients were still alive at the end of the follow-up. All BCLC stage 0 patients were alive at the end of the data analysis. The median survival values for the BCLC B and C patients were 40.7 months and 1.1 months, respectively, in our cohort.

## 4. Discussion

Chronic HBV and HCV infections are endemic and considered to be the most important risk factor for HCC in Taiwan [7,24]. HBV carriers accounted for up to 20% of the general population in previous decades in Taiwan, which brought upon an increased risk of HCC incidence [25]. However, the prevalence of chronic HBV carriers and HBV-related HCC incidence declined dramatically after a nationwide HBV immunization program for newborns in Taiwan was launched on 1 July 1984 [26]. Subsequently, the role of HCV in the etiology of HCC in Taiwan has increased in the last twenty years [7]. The previous case–control study also found that chronic HCV infection was the primary cause of excess mortality from HCC in an HBV–HCV-endemic area [27].

HCV infection has become a major public health issue in Taiwan. To uncover the asymptomatic hepatitis carriers and to eliminate hepatitis C in this population deserve more attention and efforts. There is still no effective vaccine to control HCV infection. The estimated prevalence of anti-HCV was 3.28% (1.8–5.5%) in the general population, whereas it is as high as 6% to 30% in some townships [12,17,28]. The prevalence is much higher than the global prevalence [8,29,30]. In a modeling study in 2015, the global prevalence of active HCV was estimated to be 1% [30]. The availability of short and easily tolerable treatment courses that involve direct-acting antiviral drugs has made HCV elimination more feasible than interferon-based treatment. Subsequently, this has led to viral eradication in more than 98% of patients infected with hepatitis C virus. The treatment course usually only lasts for 8–12 weeks and with no or only minor adverse effects [31]. However, there is still an extensive gap between the numbers of hepatitis patients infected and those diagnosed, especially in remote or rural townships [8]. National Health Insurance (NHI) in Taiwan has put emphasis on the public health issue. To implement a cost-effective strategy to eradicate HCV, it is important to know the trends and gain a more detailed understanding of the possible high-HCV-endemic areas. A series of geographical distributions of HCV in Taiwan were analyzed [13]. In addition, outreach screening programs were conducted to increase the accessibility of patients and to improve the health status for residents in rural communities [17]. Tien et al. conducted a program of village-by-village screening tests in the Laiyi and Mudan Townships for hepatitis, linking outreach hepatology care at the two indigenous townships in Pingtung county localized at the most southern part of Taiwan [32]. Up to 95.7% of residents with HCV in this program achieved a sustained virological response (SVR), defined as undetectable HCV viremia, for at least 12 weeks after the end of treatment [32]. Since 2018, YLCGMH has coordinated with the Nursing Department of Chiayi Chang Gung University of Science and Technology to improve villagers’ health in nearby townships [12,16,17,18,19,20]. Outreach health checkups, including liver disease screening, have been conducted in five surrounding townships. The staff of primary health care centers called back villagers when they were positive for anti-HCV or HBsAg, linking to further care [17]. Until 2022, an estimated 94.7% of patients positive for anti-HCV came back for HCV RNA testing, and 95.2% of HCV patients in these townships have been cured either with peginterferon-based therapy or direct-acting antivirals drugs. Outreach screening programs and linkage to care would be a feasible model to decrease the burden of chronic virial hepatitis for residents in rural communities.

The staff at primary health care centers are an important cadre of the primary health care workforce in many rural or low-income communities. They serve as liaisons between community members and health care providers, delivering appropriate health services to the community. The primary health care center links the villagers to medical care, but also provides appropriate health information. Many rural residents are unaware if they have chronic hepatitis C infection. In addition, very few are aware of the new drugs with high cure rates and minimal side effects that can greatly reduce their health burden [33]. Staff at the primary health care center call back villagers for HCV RNA confirmation and free DAAs treatment when they are positive for anti-HCV, providing information on the benefits of DAA and follow-up medication compliance. Furthermore, they educate and empower residents in rural settings to raise awareness about the transmission routes of hepatitis C virus infection. The services offered by the primary health care center are very essential not only for the villagers, but also for the public health system, especially to reach poorer populations and those living in remote areas with limited access to quality medical care.

Hepatocellular carcinoma has been one of the most common cancers in Taiwan for the past four decades. Nevertheless, the incidence of HCC decreased after the reimbursement of costs from the National Health Insurance (NHI) scheme for nationwide nucleos(t)ide analogues and interferon-based treatment for chronic HBV and HCV patients in 2003 and successful antiviral therapy treatment [34,35]. Furthermore, survival improved after the improvement in tumor detection and treatment instrumentation in recent decades. Curative treatments became more widely available to HCC patients, generally improving prognosis [36]. In Taiwan, the 5-year survival rates of BCLC stages 0, A, B, C, and D were 70%, 58%, 34%, 11%, and 4%, respectively [11]. The median survival years of BCLC stages 0, A, B, C, and D were 9.7, 6.3, 2.7, 0.6, and 0.2 years, respectively [11]. The proportion of BCLC stage 0 patients increased from 6.2% to 11.3%. The survival rate significantly increased year-by-year from 2011 to 2019 [11]. 

It was interesting to note that even though the overall survival rates improved, the survival rates of patients with HCC who lived in rural areas remained lower than those in urban areas [37,38]. The phenomenon was not only found among US adults, but also among patients with hepatocellular carcinoma residing in rural locations of Australia and Taiwan [38]. The survival differences might be related to the fact that urban areas in Taiwan have more medical resources and populations with higher socioeconomic statuses. On the other hand, patients from rural households may have suboptimal access to liver disease care, which may translate into worse HCC outcomes in the rural areas of Taiwan [17]. 

Mailiao and Taihsi are rural indigenous townships with a high risk of HCC mortality in the Yunlin county, with high priorities for HCC detection and treatment programs [14]. The two townships were endemic for HCV infection due to limited medical recourses, low socioeconomic status, poor hygiene practices and inadequate sanitary conditions [12,17,20]. It is served by the Chang Gung Memorial Hospital Yunlin Branch (YLCGMH), a 100-bed local hospital located near Mailiao and Taihsi. The YLCGMH is committed to providing HBV and HCV treatment for villagers living in rural areas. More than 1000 patients with HCV viremia have been detected by the annual health checkups and most of them have been treated by either interferon-based or direct-acting antiviral agent (DAA) regimens at YLCGMH. 

Among our study cohort, all the patients with detectable HBV DNA received nucelos(t)ide analogues according to international guidelines. Nowadays, the effectiveness of DAAs against HCV, following successful treatment of early hepatocellular carcinoma (HCC), has been extensively studied. The benefit of DAA against HCV following successful treatment of HCC remains controversial. One meta-analysis that assessed the HCC recurrence risk following DAA administration revealed inconclusive results [39]. A large number of studies revealed that DAA therapy was associated with a significant reduction in the risk of death, improved overall survival and reduced the risk of hepatic decompensation [40,41,42]. To achieve better overall survival rates of HCV-related HCC, most patients with detectable HCV RNA received peginterferon-based therapy or DAA for HCV eradication. The SVR of the screening and control groups was as high as 86.2% and 92.3%, respectively. 

The program of annual health checkups enabled the early detection of HCC. Patients in the screening group had better liver function reserve, demonstrated early-stage HCC and single tumors compared with the control group. In addition, patients in the screening group demonstrates more prolonged survival than the control group, with *p* = 0.048. Annual health checkups for the HCV-endemic townships can indeed identify HCC at early stages and improve patient survival in rural indigenous villages.

Medical accessibility and continuum of care are essential in the outcome of HCC treatment. However, while YLCGMH can perform almost all types of laboratory tests, in addition to CT, and MRI scans for HCC detection, it has minimal facilities for HCC treatment. To compensate for this limitation and to close the medical resources gap between rural and urban townships, a comprehensive hepatology care program was set up in a remote HCV-endemic area. Patients with advanced liver diseases, mostly HCC, were referred to a biweekly hepatology clinic established in October 2017. Patients with newly diagnosed or recurrent HCC were transferred to the CGMH Chiayi branch (CYCGMH) for further management. Furthermore, medical shuttles were provided between YLCGMH and CYCGMH. Post-HCC treatment patients were under regular surveillance in YLCGMH.

More than half of the patients with BCLC 0/A were alive at the end of the follow-up in the rural communities that were endemic for HCV. All BCLC stage 0 patients were alive at the end of the data analysis. Our cohorts’ median survival rate for patients in the B stage was 3.34 years. In Taiwan, the 5-year survival rates of BCLC stages 0 and A were 70% and the median survival rates of BCLC stage B patients were 2.7 years [11]. The expectations of the BCLC guideline 2022 for patients in stages 0/A and B are >5 and >2.5 years [9]. The outcome of BCLC 0/A and B in the rural cohort study was not inferior to the results of Taiwan and were also above the expectations of the BCLC guideline 2022 for stages 0/A and B. In consequence, the program shortened the health gap between the indigenous and the general population in Taiwan and improved patient outcomes. 

Chang Gung Memorial Hospital Yunlin Branch and the Nursing Department of Chiayi Chang Gung University of Science and Technology played an important role in improving the health status of residents living in central west coastal Taiwan, endemic for hepatitis C virus infection. Outreach screening programs and linkage to care systems for HBV treatment and HCV elimination can not only avoid the development of more advanced liver diseases, but also reduce the incidence of HCC. Furthermore, the annual health checkups and abdominal ultrasonography conducted by hepatologists resulted in the early detection of HCC. A biweekly hepatology clinic, referral system and medical shuttles provided optimal access to HCC treatment, as well as health care accessibility. The comprehensive liver disease care program provided early detection of liver tumors and improved the survival of HCC patients in a remote HCV-endemic area. 

Based on the above results, we proposed a practical model to improve the health status of rural citizens. However, there are still some limitations in our study. First, this was a retrospective study conducted in a local hospital and the sample size was small. This may limit the generalizability of our findings and the results may be affected by extreme values due to the small sample size. In addition, the screening coverage was not comprehensive because we only recruited volunteered participants instead of using a systematic screening approach. Third, the follow-up duration may not be long enough to present the real effects of the comprehensive liver disease care program. Large prospective cohort studies should be performed. 

## 5. Conclusions

HCV eradication and HCC treatment are essential issues for rural indigenous townships in central west coastal Taiwan, due to limited medical resources or settings with inadequate access to medical resources. This successful comprehensive hepatology care model in this remote endemic area showed that the screening of hepatitis and HCC and linkage to the residents’ access to high-quality care result in favorable HCC survival rates.

## Figures and Tables

**Table 1:** Clinical, laboratory and radiological features of the 77 HCC patients in the screening and control groups.

Variables	Total (*n* = 77)	Screening Group (*n* = 53)	Control Group (*n* = 24)	*p*-Value
Age, years (mean ± SD)	65.7 ± 11.1	66 ± 11.0	65.3 ± 115	*p* = 0.809
Gender				*p* = 0.994
Male, n (%)	61 (79.2%)	42 (79.2%)	19 (79.1%)	
Female, n (%)	16 (20.8%)	11 (20.8%)	5 (20.8%)	
Residence				*p* = 0.000
Mailiao/Taihsi, n (%)	66 (85.7%)	53 (100%)	13 (54.2%)	
Others, n (%)	11 (14.3)	0	11 (45.8%)	
Hepatitis				*p* = 0.523
NBNC, n (%)	8 (10.3%)	6 (11.3%)	2 (8.3%)	
HBV, (%)	15 (19.4%)	10 (18.8%)	5 (20.8%)	
HCV, n (%)	41 (53.2%)	26 (49.0%)	15 (62.5%)	
HBV + HCV, n (%)	13 (16.8%)	11 (20.7%)	2 (8.3%)	
Bilirubin (g/dL; median, IQR)	0.9 (0.6–1.2)	0.9 (0.6–1.2)	1.0 (0.6–1.3)	*p* = 0.466
Albumin (g/dL; median, IQR)	4.2 (3.9–4.6)	4.3 (4.1–4.6)	3.9 (3.4–4.6)	*p* = 0.040
AST (U/L; median, IQR)	45.9 (26.0–55.5)	37.2 (24.5–44.5)	65.2 (29–77.2)	*p* = 0.044
ALT (U/L; median, IQR)	43.5 (21.5–59.0)	39.9 (21–47.5)	51.4 (25.2–69.2)	*p* = 0.140
AFP (ng/mL; median, IQR)	8.4 (3.6–54.3)	5.5 (2.8–33.4)	17.2 (9.6–316.2)	*p* = 0.07
<20 ng/mL, n (%)	50 (64.9%)	37 (69.8%)	13 (54.1%)	
<400 ng/mL, n (%)	17 (22.0%)	12 (22.6%)	5 (20.8%)	
>400 ng/mL, n (%)	10 (12.9%)	4 (7.5%)	6 (25.0%)	
Child–Pugh grade				*p* = 0.052
A, n (%)	73 (94.8%)	52 (98.1%)	21 (87.5%)	
B, n (%)	4 (5.1%)	1 (1.8%)	3 (12.5%)	
Albumin–Bilirubib (ALBI) grade				*p* = 0.031
I	53 (68.8%)	41 (77.3%)	12 (50%)	
II	23 (29.8%)	12 (22.6%)	11 (45.8%)	
III	1 (1.2%)	-	1 (4.1%)	
Tumor number				*p* = 0.001
Single	60 (77.9%)	46 (86.7%)	14 (58.3%)	
Multiple	17 (22.1%)	7 (13.2%)	10 (41.7%)	
Tumor size				*p* = 0.113
>2 cm, n (%)	26 (33.7%)	18 (34.0%)	9 (37.5%)	
>2.1–5 cm, n (%)	46 (59.7%)	33 (62.3%)	11 (45.8%)	
>5 cm, n (%)	5 (6.4%)	2 (3.8%)	4 (16.6%)	
Barcelona Clinic Liver Cancer (BCLC) stage				*p* = 0.028
0, n (%)	21 (27.2%)	14 (26.4%)	7 (29.2%)	
A, n (%)	40 (51.9%)	32 (60.4%)	8 (33.3%)	
B, n (%)	14 (18.1%)	7 (13.2%)	7 (29.2%)	
HBV DNR (+)	21	16	5	*p* = 0.839
NUC	21	16	5	
HCV RNA (+)	45	30	15	*p* = 0.512
DAA treatment	33	23	10	
Peginterferon-based therapy	9	6	3	
No treatment	3	1	2	
Treatment effect for HCV, n	45	30	15	*p* = 0.423
SVR (+), n (%)	37 (88.1%)	25 (86.2%)	12 (92.3%)	

## Data Availability

All analyzed data are included in this published article. The original data are available upon reasonable request from the corresponding author.
